# Supplementary material for: Effects of Three Different Family-Based Interventions in Overweight and Obese Children: The “4 Your Family” Randomized Controlled Trial
Source: Nutrients. 2021 Jan 24;13(2):341. doi: 10.3390/nu13020341 (PMC7911878; doi:10.3390/nu13020341)
Supplement: Supplementary file 1 [file nutrients-13-00341-s001.pdf]

Supplementary Table S1. Overview of the topics covered in the intervention, strategies & conveyance method of each by Group.

Session	Topics	Strategies	Conveyance Method		
			Group 1	Group 2	Group 3
Session 1: Nutritional needs of children	Nutritional needs and goals	Overview of health outcomes	Trained Dietitian	Trained Dietitian throughout	Video by same as Group 1 dietitian
Session 2: Managing problematic behaviors	Increasing children's self-esteem	Discussing the encountered barriers and the possible resolutions	Psychologist		Video by same as Group 1 psychologist
Session 3: Practice for success	Readiness to change	Setting goals	Psychologist		Video by same as Group 1 psychologist
Session 4: Understanding food pyramid	Food groups and daily servings	Eating a healthy balanced diet	Trained Dietitian		Video by same as Group 1 dietitian
Session 5: Exercise: a friend for your health	Physical activity goals, children's participation in sport	Live fitness show designed for school aged children	Delivered by physical education teacher		Video by same as Group 1 physical education teacher
Session 6: Time for physical activities	Providing active daily alternatives such as taking the stairs; Improving movement skills	Encourage a minimum of 20 min per day physical activity	Delivered by physical education trainer		Video by same as Group 1 physical education teacher
Session 7: Importance of breakfast	Establishing healthy eating routines	Prioritizing breakfast	Trained Dietitian		Video by same as Group 1 dietitian
Session 8: Importance of eating fruits & vegetables	Encouraging healthy eating behavior	Increase the amount of fruit and vegetables at home	Trained Dietitian		Video by same as Group 1 dietitian
Session 9: Time for cooking	Creating and selecting healthy meals & snacks	Live cooking show	Chef		Video by same as Group 1 Chef
Session 10: Healthy recipes	Modifying child friendly recipes for a more healthy but tasty composition	Creatively involving children in new food preparation & consumption	Chef		Video by same as Group 1 Chef (the chef encourages parents to involve their child when preparing specific dishes)
Session 11: Labels, Fast food & meals away from home	Reducing fat and sugar intake, food labels	Consume fewer processed food and providing examples on how to read labels	Trained Dietitian		Video by same as Group 1 dietitian
Session 12: Importance of family meal	Family eating tips	Planning and preparing family meals	Trained Dietitian		Video by same as Group 1 dietitian

Group1: on site Group based intervention for children and parents; Group 2: interpersonal face- sessions with highly skilled and trained dietitians; Group 3: homebased specifically created website intervention. All session were conveyed by the dietitian that the child was enrolled too for children in Group 2 and in Group 3, the information was conveyed by specific web developed pages per topic. All health care workers were trained and given specific outlines to follow and questionnaires to complete. Questionnaires were also given to the participants to evaluate understanding and assess health care worker compliance.
